# Nasal self-invasive behavior as an unnoticed behavioral oddity in Schizophrenia: A rare case report

**DOI:** 10.1192/j.eurpsy.2026.12118

**Published:** 2026-07-31

**Authors:** H. Zizi, R. Rania, Z. Ennaciri, I. Adali, F. Manoudi

**Affiliations:** Ibn Nafis Hospital, Psychiatry, Mohammed VI university hospital, Marrakech, Morocco

## Abstract

**Introduction:**

Behavioral oddities represent a frequently overlooked dimension of schizophrenia, particularly when they involve the patient’s own body. Silent self-invasive acts, underpinned by delusional rationalization, may remain unnoticed in the absence of functional complaints. We report an unusual case of incidental discovery of bony foreign bodies in the nasal cavities of a patient with schizophrenia, in the context of covert self-injurious behavior.

**Objectives:**

The objective of this case report is to highlight an unusual form of silent self-injurious behavior in schizophrenia, consisting of nasal

self-invasion with osseous fragments. This observation aims to draw attention to the clinical relevance of systematic somatic assessment in chronic psychotic patients, even in the absence of functional complaints, and to emphasize the importance of integrating psychopathological interpretation with medical imaging findings.

**Methods:**

This work is based on a single clinical case observed in a 29-year-old male patient with schizophrenia. Data were collected from direct clinical observation during psychiatric hospitalization, retrospective patient interviews, and systematic brain computed tomography (CT) performed during routine assessment. A follow-up CT scan was obtained to confirm the spontaneous extraction of the foreign bodies. Clinical data were complemented by a psychopathological analysis of the patient’s discourse and behavior.

**Results:**

A 29-year-old man, followed for schizophrenia for three years, was admitted for acute psychotic relapse with auditory hallucinations. A routine brain CT scan unexpectedly revealed two foreign bodies occupying the nasal cavities, tubular in shape and osseous in nature, measuring 8 × 0.5 cm and 7.5 × 0.5 cm, respectively. On retrospective inquiry, the patient acknowledged self-insertion of these bone fragments with the aim of blocking voices. He manually extracted them during the clinical interview, while the remaining fragment was spontaneously expelled during a sneezing episode. No pain or functional impairment was reported. A control CT confirmed patent nasal cavities with mild, regular reactive thickening.

This case illustrates a self-inflicted bodily oddity rationalized by delusional ideation. Comparable reports are scarce in the literature and typically involve heterogeneous objects, in a context of altered body schema and disturbed ego boundaries. Beyond its clinical singularity, this observation underscores the need for systematic somatic assessment in chronic psychotic patients.

**Image 1:**
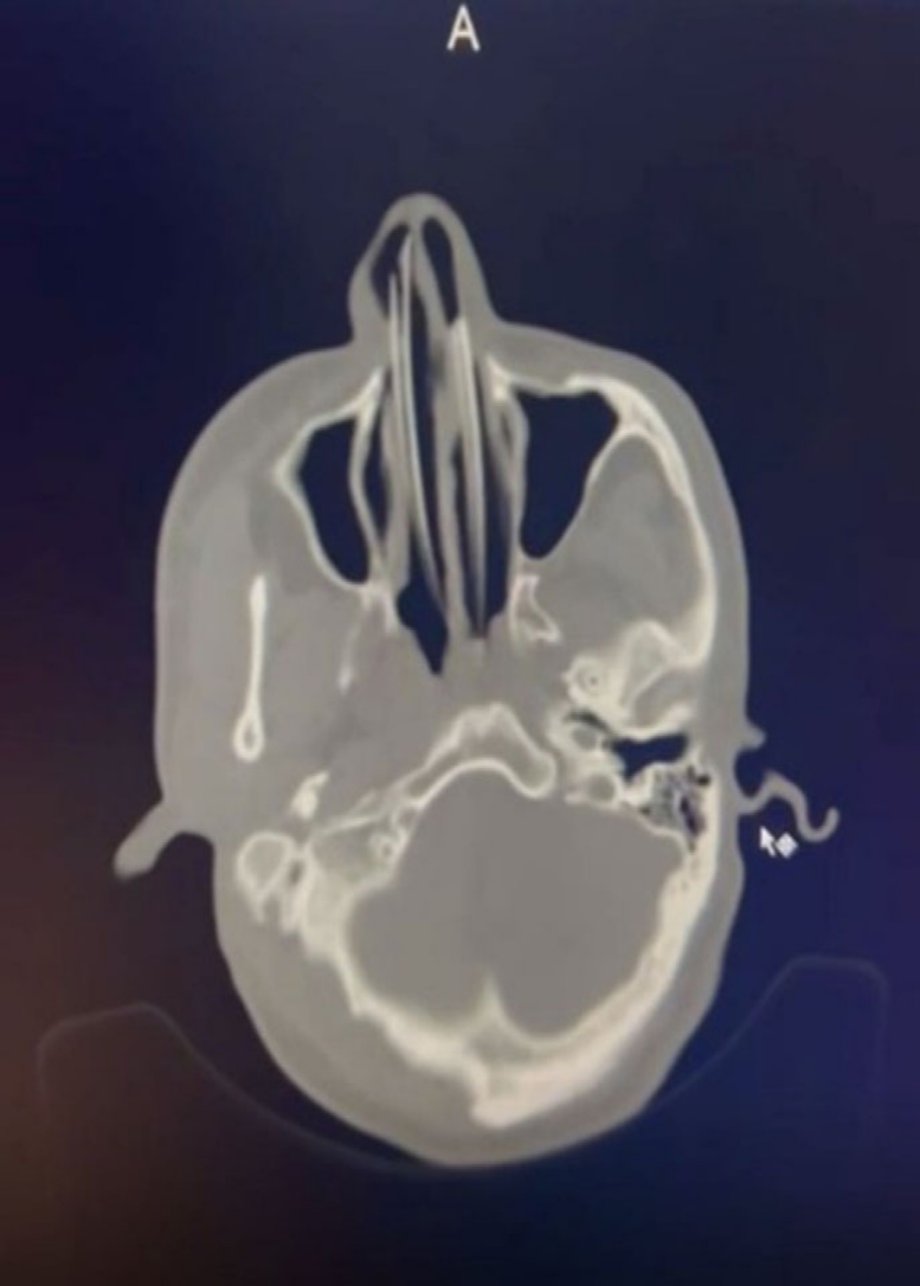
Image 1: Long description.

**Image 2:**
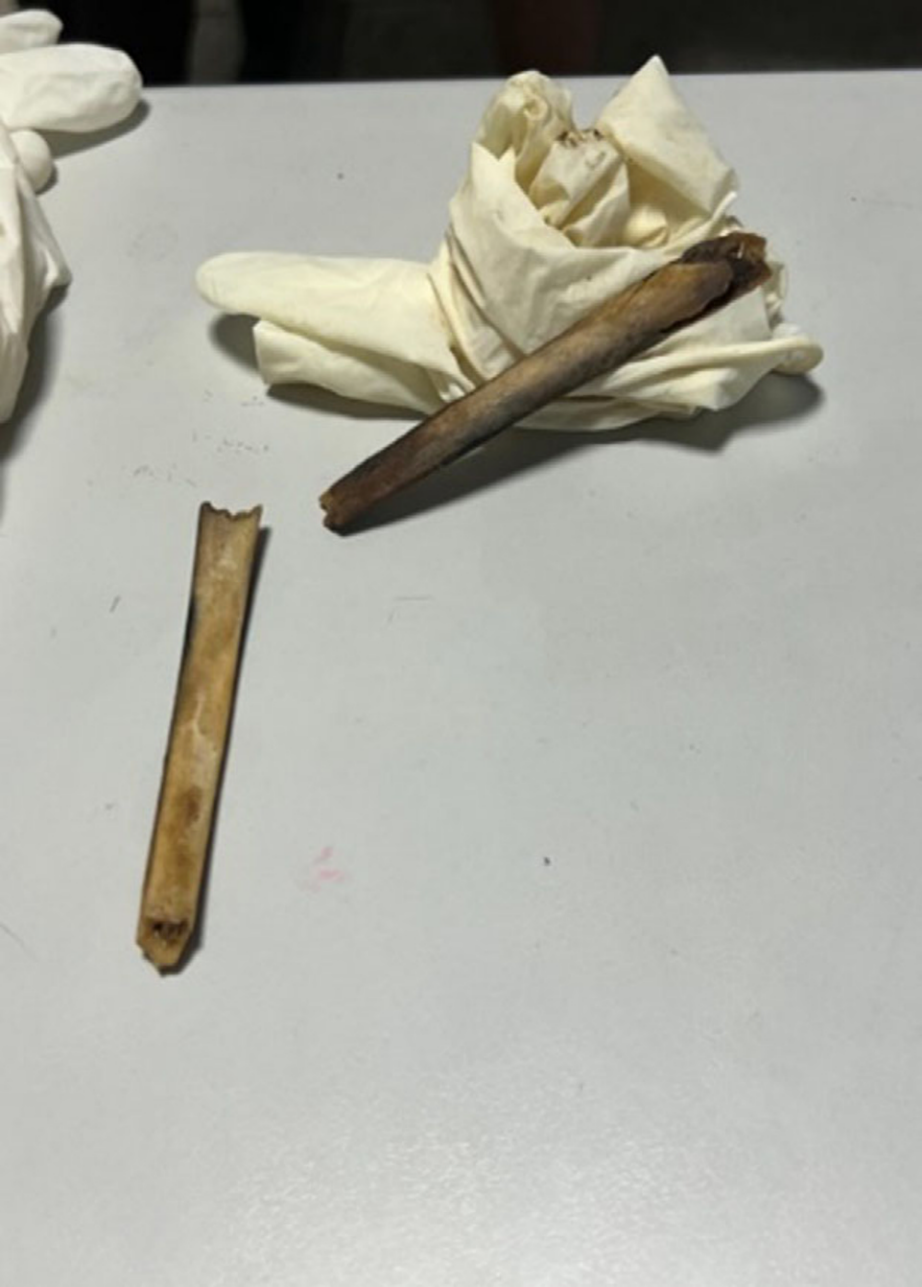

**Image 3:**
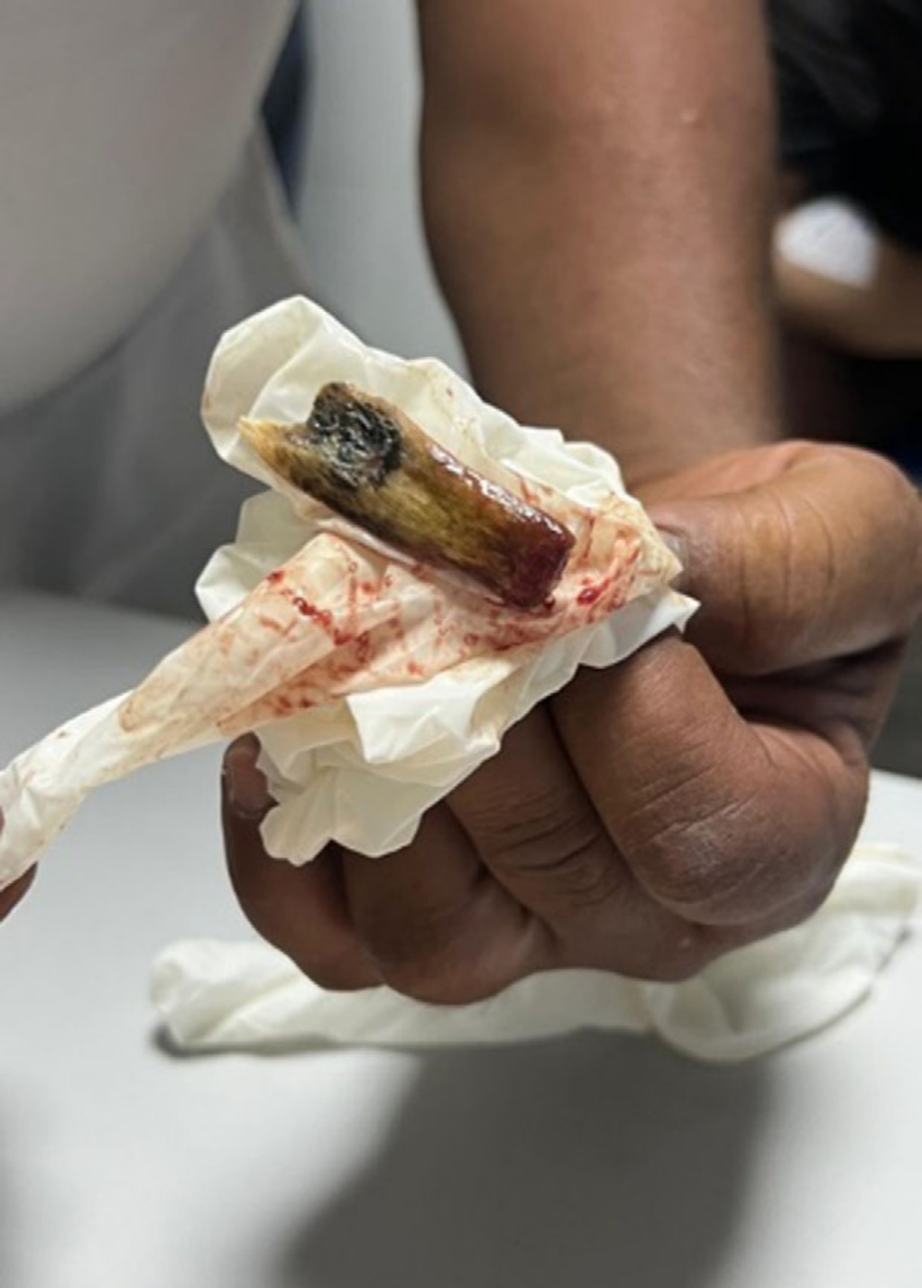

**Conclusions:**

This case emphasizes the relevance of thorough somatic evaluation and targeted inquiry in psychiatric practice to detect overlooked self-invasive behaviors. An integrative approach, combining clinical observation, imaging, and psychopathological analysis, is essential to understand and prevent atypical bodily acts in chronic psychosis.

**Disclosure of Interest:**

None Declared

## Long descriptions

### Image 1: Long description

This axial C T scan displays the following anatomical structures from top to bottom.

* At the top anterior position is the nasal region, featuring the nasal septum and turbinates within the nasal cavity.

* Moving laterally from the midline are the maxillary sinuses, appearing as dark, air-filled pockets.

* In the center of the skull base, the sphenoid bone and associated structures are visible.

* On the right side of the image, the external auditory canal and the mastoid air cells are clearly defined with a porous, honeycomb-like bone texture.

* A small, curved external structure is visible on the far right lateral edge, likely representing the pinna of the ear.

* At the bottom posterior position, the large opening of the foramen magnum is visible, surrounded by the occipital bone.

* A single capital letter A is centered at the very top of the frame, indicating the anterior orientation.

Navigate back to Image 1:.

